# Gender differences in the regulation of MLC_20_ phosphorylation and smooth muscle contraction in rat stomach

**DOI:** 10.3892/br.2019.1252

**Published:** 2019-11-04

**Authors:** Othman A Al-Shboul, Ahmed N Al-Dwairi, Mohammad A Alqudah, Ayman G Mustafa

Biomed Rep 8:283-288, 2018; DOI: 10.3892/br.2018.1053

After the publication of the above paper, the last-listed author, Ayman Mustafa, realized that his present address (Qatar University, Doha, Qatar) should have been included in the correspondence information for the manuscript. Therefore, the authors’ names and affiliations for this paper should have been presented as follows:

OTHMAN A. AL-SHBOUL^1^, AHMED N. AL-DWAIRI^1^, MOHAMMAD A. ALQUDAH^1^ and AYMAN G. MUSTAFA^2,3^

Departments of ^1^Physiology and Biochemistry, and ^2^Anatomy, Faculty of Medicine, Jordan University of Science and Technology, Irbid 22110, Jordan

*Correspondence to*: Dr Othman A. Al-Shboul, Department of Physiology and Biochemistry, Faculty of Medicine, Jordan University of Science and Technology, Irbid 22110, Jordan

E-mail: oashboul@just.edu.jo

*Present address*: ^3^Department of Basic Medical Sciences, College of Medicine, Qatar University, Doha, Qatar

The authors regret this oversight in terms of their author affiliations, and apologize for any inconvenience caused.

